# Gut microbiota and fecal volatilome profile inspection in metabolically healthy and unhealthy obesity phenotypes

**DOI:** 10.1007/s40618-024-02379-2

**Published:** 2024-06-21

**Authors:** F. M. Calabrese, V. A. Genchi, N. Serale, G. Celano, M. Vacca, G. Palma, M. Svelto, L. Gesualdo, M. De Angelis, F. Giorgino, S. Perrini

**Affiliations:** 1 Department of Soil, Plant and Food Sciences, University of Bari Aldo Moro, Bari, Italy; 2 Section of Internal Medicine, Endocrinology, Andrology and Metabolic Diseases – Department of Precision and Regenerative Medicine and Ionian Area, University of Bari Aldo Moro, Bari, Italy; 3 Department of Biosciences, Biotechnologies and Environment, University of Bari Aldo Moro, Bari, Italy; 4 Nephrology, Dialysis and Transplantation Unit– Department of Precision and Regenerative Medicine and Ionian Area, University of Bari Aldo Moro, Bari, Italy

**Keywords:** Metabolically healthy obesity, Metabolically unhealthy obesity, Bariatric surgery, Metabolomics, Restrictive caloric diet

## Abstract

**Background:**

People with metabolically healthy (MHO) and metabolically unhealthy obesity (MUO) differ for the presence or absence of cardio-metabolic complications, respectively.

**Objective:**

Based on these differences, we are interested in deepening whether these obesity phenotypes could be linked to changes in microbiota and metabolome profiles. In this respect, the overt role of microbiota taxa composition and relative metabolic profiles is not completely understood. At this aim, biochemical and nutritional parameters, fecal microbiota, metabolome and SCFA compositions were inspected in patients with MHO and MUO under a restrictive diet regimen with a daily intake ranging from 800 to 1200 kcal.

**Methods:**

Blood, fecal samples and food questionnaires were collected from healthy controls (HC), and an obese cohort composed of both MHO and MUO patients. Most impacting biochemical/anthropometric variables from an a priori sample stratification were detected by applying a robust statistics approach useful in lowering the background noise. Bacterial taxa and volatile metabolites were assessed by qPCR and gas chromatography coupled with mass spectrometry, respectively. A targeted GC–MS analyses on SCFAs was also performed.

**Results:**

Instructed to follow a controlled and restricted daily calorie intake, MHO and MUO patients showed differences in metabolic, gut microbial and volatilome signatures. Our data revealed higher quantities of specific pro-inflammatory taxa (i.e., *Desulfovibrio* and *Prevotella* genera) and lower quantities of *Clostridium coccoides group* in MUO subset. Higher abundances in alkane, ketone, aldehyde, and indole VOC classes together with a lower amount of butanoic acid marked the faecal MUO metabolome.

**Conclusions:**

Compared to MHO, MUO subset symptom picture is featured by specific differences in gut pro-inflammatory taxa and metabolites that could have a role in the progression to metabolically unhealthy status and developing of obesity-related cardiometabolic diseases. The approach is suitable to better explain the crosstalk existing among dysmetabolism-related inflammation, nutrient intake, lifestyle, and gut dysbiosis.

**Supplementary Information:**

The online version contains supplementary material available at 10.1007/s40618-024-02379-2.

## Introduction

Obesity is a worldwide spread chronic condition characterized by an unbalanced between energy intake and disposal which leads to an excessive adipose tissue expansion as well as to develop a low-grade inflammation state and long-term metabolic consequences. The incidence of obesity is continuously growing and the pathology will affect one adult out of five in 2025 [1]. In this condition, the limited expandability of adipose tissue leads to increased fat accumulation in both visceral and ectopic compartments thus increasing the risk of metabolic disorders including coronary heart disease, hypertension and stroke, cancer, type 2 diabetes (T2D), dyslipidaemia [2]. Although some critical biochemical pathways dedicated to macronutrients utilization and autoimmune or inflammatory response [3] are impaired under obesity, not all obese patients develop inflammation and metabolic derangements. Indeed, recent findings describe two different phenotypes of obesity: metabolically healthy obesity (MHO), characterized by the absence of metabolic alterations, and metabolically unhealthy obesity (OB), characterized by the presence of visceral adiposity in association with least two co-morbidities [4].

Obesity is a multifactorial disease whose onset and progression are often associated with a reduction in gut alpha-diversity as well as changes in specific species abundances. Different studies reported that *eubiosis* is necessary for the maintenance of energy balance and lipid deposition in adipose tissues [5]. Indeed, in the presence of intestinal dysbiosis a pronounced energy absorption and accumulation were observed which in turn result in weight gain [6]. The main microbiota-derived regulators of energy balance and bodyweight are the short-chain fatty acids (SCFAs) which control satiety or hunger feelings by promoting the release of peptide YY (PYY), ghrelin, insulin, and glucagon-like peptide 1 (GLP-1), all factors directly involved in the regulation of appetite, food intake, and insulin secretion [7]. SCFAs also affect the intestinal functionality by regulating the tight junction and mucin layer, and may lead to chronic inflammation and metabolic dysfunction at long-term [8]. Furthermore, these compounds have been previously correlated with clinical [9] (e.g., transaminase, HbA1c, cholesterol) and anthropometric parameters, including BMI or weight, in presence of dysmetabolic conditions and inflammation [10].

Nowadays, differences in gut microbiota and fecal metabolites in MHO and MUO individuals were not deeply explored yet. Undoubtedly, targeting VOC profiles can help in elucidating the crosstalk among dysmetabolism-related inflammation, nutrient intake, lifestyle, and gut dysbiosis [8]. At this aim, we investigated clinical and dietary parameters, gut microbiota and fecal volatilome, in subjects affected by MHO, MUO and in healthy controls (HC). For the first time, our study highlighted statistically significant differences in metabolic, taxonomic and volatilome signature between MUO and MHO patients who underwent the same restrictive caloric intake.

## Methods

### Study groups

A cross-sectional prospective observational study based on subjects affected by metabolically healthy obesity (MHO) and metabolically unhealthy obesity (MUO) was here conducted. More precisely, MHO and MUO subsets were composed of 18 and 27 patients, respectively. A group of 12 healthy control subjects (HC) was included. All volunteers agreed to undergo a comprehensive clinical evaluation based on the protocol approved by the Independent Ethical Committee (IEC). The study and the informed consent process were carried out in accordance with the ethical principles of the Declaration of Helsinki, the Regulation (EU) 2016/679, the Legislative Decree 101/2018, the International Ethical Guidelines for Biomedical Research involving subjects CIOMS-WHO (2016), and the Recommendation on Research on Biological Materials of Human Origin Rec (2006). Subjects were recruited from a clinical center located at University-Hospital Consortium Polyclinic of Bari—Department of Precision and Regenerative Medicine and Ionian Area, Section of Internal Medicine, Endocrinology, Andrology and Metabolic Diseases.

**Fig. 1:**
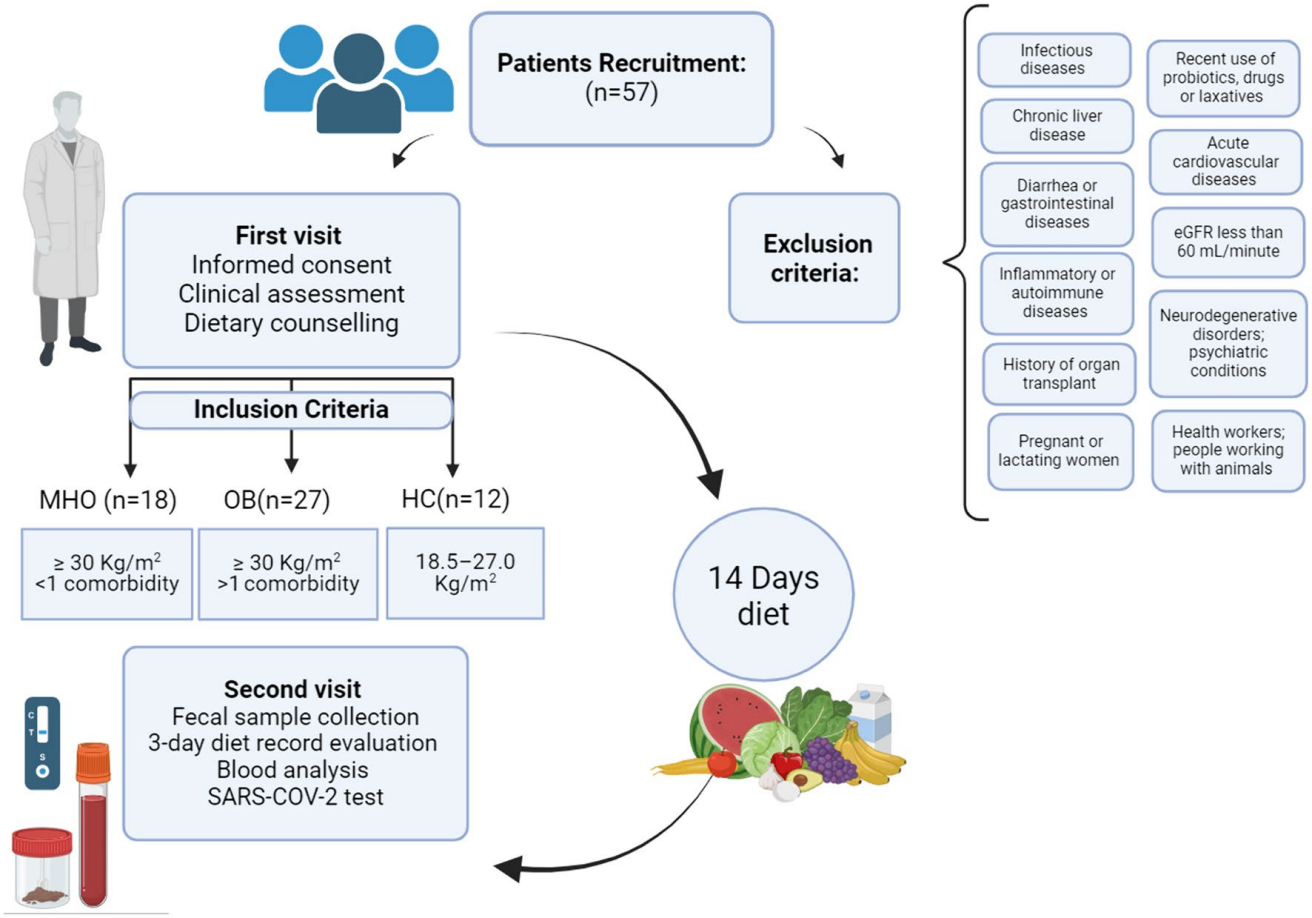
Experimental design. A cross-sectional prospective observational study was performed in three different cohorts: healthy (HC), metabolically healthy obesity (MHO), and metabolically unhealthy obesity (MUO). During the first visit, subjects were addressed to clinical assessment and hypocaloric diet (800–1200 kcal). After 14 days, patients underwent to a second visit for SARS-COV-2 test, blood analysis and fecal samples collection

HC subjects were enrolled according to the following inclusion criteria: 18–70 years old, BMI range between 18.5 and 27.0 kg/m^2^, adherence to an omnivorous diet. Subjects with obesity followed the same age and diet criteria but presented a BMI ≥ 30 kg/m^2^. Moreover, MUO individuals were characterized by other comorbidities including insulin-resistance, prediabetes or T2D, dyslipidaemia, cardiovascular diseases (hypertension), or respiratory diseases (obstructive sleep apnoea). On the other hand, as defined, MHO patients were featured by only one or no comorbidity cardio-metabolic or respiratory diseases [4]. Furthermore, among the exclusion criteria we considered: the suffering from current or previous infectious diseases (caused by HAV, HBV, HCV, HIV, Cytomegalovirus, and Epstein-Barr virus), chronic liver disease, history of *Clostridium difficile* infection, recent pharmacological therapy (3 months before the study), immunosuppressant therapy, chemotherapy, chronic therapy with proton pump inhibitors, recent use of probiotics (3 months before the study), use of laxatives, drugs or supplements, history of organ transplant, recent appearance of diarrhea, chronic diarrhea, chronic constipation, previous gastrointestinal surgery (e.g. gastric bypass), or recurrent urinary tract infections (three cases per year). Moreover, in compliance with the above mentioned protocol, study participants must not have suffered from acute cardiovascular diseases (myocardial infarction, stroke), arterial hypertension or chronic gastrointestinal diseases or systemic inflammatory diseases, did not have an estimated Glomerular Filtration Rate (eGFR) less than 60 mL/minute, did not have a diagnosis of nephropathy, or previous history of malignancy (< 5 years), autoimmune diseases or a history of chronic and systemic autoimmune disorders, and neurodegenerative disorders with psychiatric conditions. In addition, pregnant or lactating women, health workers, and anyone working with animals were excluded from the study. In addition to the inclusion/exclusion criteria listed here, all enrolled subjects had a diet regimen ranging from 800 to 1200 kcal in terms of average daily intake (Fig. 1). This was useful in balancing the effect of diet on gut microbiota metabolism on different subjects. Considering that generally healthy subjects have a higher energy metabolism than MHO and MUO patients [11], who have a lifestyle that is associated with sedentary lifestyle, we also considered as inclusion criteria a calorie intake threshold of 1000 kcal/day. The threshold was used to stratify HC individuals versus patients with obesity.

### Clinical biochemical analysis

At first medical examination, volunteers provided their signed informed consent, they filled out an anamnestic questionnaire, and they were subjected to the following procedures: evaluation of inclusion and exclusion criteria, ID assignment, and medical history evaluation. Kit for fecal samples collection were delivered to eligible participants. Fecal samples were collected in two devices with RNAlater™ Stabilization Solution (Sigma-Aldrich), ratio 1:1. One sample was used both to carry out analysis of volatile profile using Gas Chromatography coupled with Mass Spectrometry (GC–MS) and to characterize the microbiota composition based on quantitative PCR technique. In addition, participants received instructions on how to register their dietary intake by completing a 3-day food questionnaire, including a weekend day.

During the second examination, participants delivered fecal samples and provided the completed 3-day diet record. In addition, the following procedures were executed: blood test analyses, evaluation of new signs or symptoms (filling a new amnestic questionnaire), and molecular test for SARS-COV-2 (Fig. 1). Biological material from suitable volunteers was used for biobank stocking and for experimental research purposes. Medical centers stocked fecal aliquots at − 80 °C and provided for their transfer in dry ice to the Department of Soil, Plant and Food Science (DiSSPA) at University of Bari.

### Food questionnaire analysis

To avoid exogenous confounding factors that could have affected the composition of the microbiota, 14 days before recruitment all subjects were invited to have a dietary caloric intake of approximately 1000 kcal. Both MUO and MHO patients showed high compliance to diet as they were candidate for bariatric surgery requiring a consolidated pre-operative weight loss through a very low calorie diet. In addition, to investigate dietary habits, subjects have drawn up a three-day food questionnaire (two working days and one in the weekend of the second week). Food questionnaires were analysed using the WinFood^®^ software. Averages of diet nutritional values (daily caloric intake, macronutrients, and micronutrients) were returned as outputs.

### Fecal volatilomics, real time PCR, and statistical analyses

Details relative to fecal volatilome analysis, and real time PCR as well as statistical methods used have been included within the Online Supporting Information.

## Results

### Subject characteristics

According to the adopted inclusion criteria, 12 HC, 18 MHO, and 27 MUO subjects were enrolled in our cross-sectional prospective observational study. Patient anthropometric measurements/descriptions together with biochemical gathered parameters were reported in Table 1.

**Table 1:** Group comparison of anthropometric and biochemical variables featuring the 57 enrolled participants

Measured parameter	HC (n = 12)	MHO (n = 18)	MUO (n = 27)	MHO vs. HC	MUO vs. HC	MHO vs. MUO
Age (years)	44 ± 13	32 ± 11	53 ± 10	n.s	n.s	n.s
Females: Males	9:3	14:4	15:14	–	–	–
Weight (Kg)	72.15 ± 11.89	101.52 ± 13.92	109.01 ± 21.52	0.000052	0.000001	n.s
BMI	25.37 ± 2.38	37.84 ± 5.712	39.02 ± 6.06	0.000001	< 0.000001	n.s
Biochemical parameters						
White blood cells (10^3^/L)	5.53 ± 1.59	6.86 ± 1.93	7.8 ± 2.16	n.s	0.010206	n.s
Platelets (10^3^/L)	203.92 ± 28.11	250.67 ± 70.43	254.59 ± 85.45	n.s	0.045552	n.s
Insulin (µU/mL)	10.37 ± 6.43	12.47 ± 8.33	21.66 ± 13.09	n.s	0.009860	n.s
HbA1c (%)	5.15 ± 1.85	5.22 ± 0.35	6.22 ± 1.39	n.s	n.s	0.026480
25-OH Vitamin D (ng/mL)	30.92 ± 10.88	22.44 ± 7.60	18.76 ± 8.45	n.s	0.026382	n.s
hsCRP (mg/L)	2.59 ± 0.85	6.33 ± 5.16	5.86 ± 4.49	n.s	0.009860	n.s
ESR (mm/h)	12.48 ± 10.57	26.78 ± 17.68	26.77 ± 20.15	n.s	0.040768	n.s
CRP (mg/g)	40.58 ± 44.81	86.23 ± 47.68	102.95 ± 72.09	n.s	0.020526	n.s
GFR (mL/min)	92.75 ± 12.68	110.83 ± 19.16	91.69 ± 16.18	n.s	n.s	0.030379
HOMA-IR	1.76 ± 1.44	2.47 ± 1.63	5.61 ± 3.39	n.s	0.000218	0.008094
A/G ratio (%)	1.52 ± 0.19	1.46 ± 0.19	1.23 ± 0.45	n.s	0.040768	n.s
Alpha 2%	8.94 ± 1.03	9.92 ± 1.08	9.26 ± 3.52	0.019745	n.s	n.s
Beta 1%	6.00 ± 0.61	6.51 ± 0.74	5.72 ± 2.03	0.04906	n.s	n.s
ACR (mg/g)	6.75 ± 6.70	11.61 ± 9.83	23.17 ± 31.61	n.s	0.011994	n.s
Albumin %	60.11 ± 2.99	59.18 ± 3.33	51.59 ± 18.09	n.s	0.019702	0.035324
Total cholesterol (mg/dl)	183.08 ± 30.09	170.56 ± 26.70	191.52 ± 36.44	n.s	0.038456	0.028301
TG (mg/dl)	91.00 ± 42.93	91.28 ± 39.79	132.97 ± 63.39	n.s	0.020065	0.008126
ALT (U/L)	29.25 ± 13.26	28.83 ± 11.01	43.24 ± 25.25	n.s	0.026549	0.010287
AST (U/L)	20.58 ± 5.40	18.83 ± 5.98	24.86 ± 13.05	n.s	n.s	0.037268
Calcemia (mg/dl)	9.00 ± 0.40	14.43 ± 22.61	9.30 ± 0.35	n.s	0,035551	n.s
TSH (mUI/l)	1.41 ± 0.57	1.81 ± 0.97	2.60 ± 3.01	n.s	0,047811	n.s
FBG (mg/dl)	100.17 ± 55.63	92.06 ± 48.44	123.72 ± 54.67	n.s	n.s	0,044767

Statistically significant corrected P-values (q-value < 0.05) were reported for healthy controls (HC), metabolically healthy obesity (MHO), and metabolically unhealthy obesity (MUO)

*n.s.* not statistically significant

Welch’ T-test corrected by Benjamini-Hochberg

Participants had an average age ranging between 32 and 53 years. No significant differences in age distribution have been observed between both obesity phenotypes even though MUO group appears to have an older age trend as compared to MHO. Nevertheless, as indicated by the high standard deviation in each group, a high heterogeneity marked the age values. In addition, both MUO and MHO had a similar disease course length, considering that we enrolled patients suffering from obesity up to 5 years. After performing a two-group corrected statistical comparison, weight and BMI values were significantly higher in MHO and MUO subjects as compared to HC (Table 1).

Minor significant differences emerged when biochemical parameters from HC and MHO subjects were compared, i.e., Alpha2 and Beta1 globulins, resulted higher in MHO patients. Interestingly, some parameters differentiated the two obese patient subsets. In detail, the MUO subset reported significant higher levels of glycated haemoglobin (HbA1c), homeostatic model assessment for insulin resistance (HOMA-IR) index, total cholesterol, triglycerides, AST and ALT aminotransferases, and fasting blood glucose (FBG), whereas MHO showed higher level of GFR and albumin. Noteworthy, as compared to HC, MUO exhibited statistically significant differences dealing with the quantity of white blood cells, platelets, insulinemia, C-reactive protein (CRP), high-sensitivity CRP (hs-CRP), erythrocyte sedimentation rate (ESR), HOMA-IR index, albumin/creatinine ratio (ACR), triglycerides, AST and ALT aminotransferases, calcemia, and thyroid-stimulating hormone (TSH), all parameters with higher levels in MUO subjects. On the contrary, 25-OH Vitamin D, albumin/globulin ratio (A/G ratio), and albumin resulted lower in MUO as compared to HC.

Next, to highlight the most impacting variables which could influence the stratification of the analysed samples, a pattern matrix analysis was performed.

Thus, based on Rotated factor loadings the total set of 16 identified latent variables was reduced into three factors that can be attributed to different processes/profiles (Table 2). We chose to discuss those variables with loading greater than 0.5 and with uniqueness lower than 0.5, where 1 represents commonality. Due to the heterogeneous nature of measured parameters, a mixture of hormonal, anthropometric and biochemical variables constitute each identified factor.

**Table 2:** Rotating factor analysis

Variable	Factor 1	Factor 2	Factor 3	Uniqueness
Platelets	0.020095692	− 0.006823868	− 0.143468649	0.978965774
Blood pressure max	0.061680614	0.031880586	− 0.154234404	0.971390514
Sodium level	0.024911039	0.12691395	0.192898503	0.946062463
Height	0.110312003	0.069867718	0.258791326	0.915976841
Prolactin	0.329965753	− 0.079092401	− 0.050967228	0.882269309
Low Density Lipoprotein (LDL)	0.213345705	0.316398074	0.046306148	0.852231636
Total cholesterol	0.231882061	0.334525235	− 0.046089413	0.832199428
Waist	0.364994359	0.197207608	0.024045799	0.827310129
Hip circumference	0.38878113	0.127930546	− 0.240842404	0.774477962
Ab anti-thyroid perossidase	0.418297357	0.206367851	0.130834169	0.765321895
Glomerular Filtration Rate (GFR)	0.449347231	0.161561318	0.213121998	0.72656419
Estradiol	0.37482969	0.375919233	0.140103081	0.698558562
Mean Cell Volume (MCV)	0.316237991	0.421257348	0.301381256	0.631705299
Free triiodothyronine (FT3)	0.402248408	0.391959959	0.23405361	0.62978252
Ab anti-thyroglobulin (Tg)	0.505983992	0.279816214	0.202535778	0.52466224
Neutrophil	0.459466277	0.40013959	0.065301084	0.524514976
Free thyroxine (FT4)	0.524283689	0.195960908	0.250676163	0.523887385
Cortisol	0.479455275	0.34704698	0.161542799	0.523584936
Thyroid-Stimulating Hormone (TSH)	0.518137929	0.329437713	0.041915164	0.521246849
Adrenocorticotropic hormone (ACTH)	**0.543950727**	0.218508551	0.238246189	0.49961036
Mean Corpuscolar Hemoglobin (MCH)	0.27113476	0.45685934	0.367243554	0.48289772
Alpha2	0.460464854	0.455178183	0.0942716	0.57189785
Fasting blood glucose (FBG)	0.530987473	0.308362909	0.227312354	0.571293658
Lymphocyte	0.489657104	0.216128886	0.394077017	0.558227661
25-hydroxyvitamin D-(comment)	0.578955769	0.212962766	0.265027199	0.549217788
Thyroglobulin (Tg)	0.211608977	0.583877511	0.257377174	0.548065766
Blood pressure min	0.518785104	0.401986482	0.147257659	0.547584098
Azotaemia	0.54723218	0.278432113	0.277594161	0.545954017
High Density Lipoprotein (HDL)	0.634011345	0.177344281	0.186959288	0.531624925
Indirect bilirubin	0.496099371	0.382196987	0.288535908	0.524557914
Eosinophil	0.529331028	0.293902804	0.353474867	0.508485335
Mean Corpuscular Haemoglobin Concentration (MCHC)	0.303111274	0.444232177	0.450468694	0.507859274
Luteinizing Hormone (LH)	**0.659798074**	0.14223388	0.210363666	0.500183193
Testosterone free	0.346195358	0.471255201	0.399104493	0.498782914
Monocyte	0.450601431	0.421646381	0.353751532	0.494032546
Alpha1	**0.557619568**	0.416021602	0.151973612	0.492890439
Weight	0.495531656	**0.511283908**	0.028500857	0.492224891
Blood creatinine	0.303318788	**0.527356532**	0.385348248	0.481399517
White Blood Cells (WBC)	0.352471567	**0.627095406**	0.033513668	0.481391978
Follicle Stimulating Hormone (FSH)	**0.691620908**	0.112467322	0.177897866	0.477364012
Phosphoremia	0.491259396	0.21538272	0.361812699	0.461189074
Total bilirubin	0.437219104	0.421601609	0.299400741	0.444006922
Beta1	**0.662410933**	0.230764223	0.262207908	0.43920665
Potassium	0.480704357	0.41139375	0.403826321	0.436602807
Body Mass Index (BMI)	**0.638141851**	0.396920708	− 0.051392926	0.432587561
high-sensitive C-reactive protein (hsCRP)	**0.645547973**	0.397228173	0.042400583	0.423679789
C-reactive protein (CRP)	**0.749648038**	0.541111586	0.021378968	0.48448761
Gamma	**0.716512308**	0.26472573	− 0.07426934	0.411014469
Glycosylated haemoglobin (HbA1c)	**0.595368172**	0.451078623	0.215050788	0.395817968
Red Cell Distribution Width (RDW)	**0.736289398**	0.24934438	0.066482627	0.391285376
Direct bilirubin	**0.546131733**	0.427911857	0.360293684	0.388820045
Calcemia	**0.614020887**	0.400484567	0.288988374	0.379076168
Albumin/creatinine ratio (ACR)	**0.617816129**	0.482949591	0.090491501	0.376874199
Albuminemia	0.477237218	0.4539142	0.452838988	0.361143399
Alanine aminotransferase (ALT)	0.335693297	**0.598004787**	0.429506374	0.345224565
Aspartate aminotransferase (AST)	0.434060796	**0.559883262**	0.394005843	0.342881362
Erythrocyte Sedimentation Rate (ESR)	**0.750585436**	0.281870714	− 0.122691984	0.342117063
Protein total	0.412550035	0.490380723	0.219438	0.336156163
Gamma-glutamyl transferase (GGT)	0.234698335	**0.725816018**	0.314578918	0.319147896
Basophil	0.440935995	0.380315083	0.513066052	0.299511915
Waist-to-height ratio (WHR)	0.488892543	0.464936086	0.100477583	0.29916576
HOMA for Insulin Resistance	0.362257006	**0.761572191**	− 0.027294153	0.288032662
Total testosterone (TT)	0.13260411	**0.625486494**	0.555447133	0.282661176
Beta_2	**0.638638417**	0.564029182	− 0.023581338	0.273456077
Insulin	0.35588476	**0.783136334**	− 0.047629641	0.25777496
Red Blood Cell (RBC)	0.219544828	**0.74702247**	0.322219077	0.247811387
Haematocrit (HCT)	0.099569856	**0.894564444**	0.352184825	0.065806141
Haemoglobin (HGB)	0.06613859	**0.904076676**	0.411750332	0.008732716
Albumin	0.275814219	0.176625158	**0.943078251**	0.005
Ratio ANAG	0.302030598	0.200303945	**0.930374992**	0.005

Variable common variance associated with factors. Three factors out of 16 latent variables were chosen based on eigenvalue (> 2). Factor loadings greater than 0.5 and with a uniqueness lower than 0.5 i.e. most impacting on the relative factor are reported in bold. Uniqueness values that tend to 1 indicates commonality

### Discriminant analysis of principal components

**Fig. 2:**
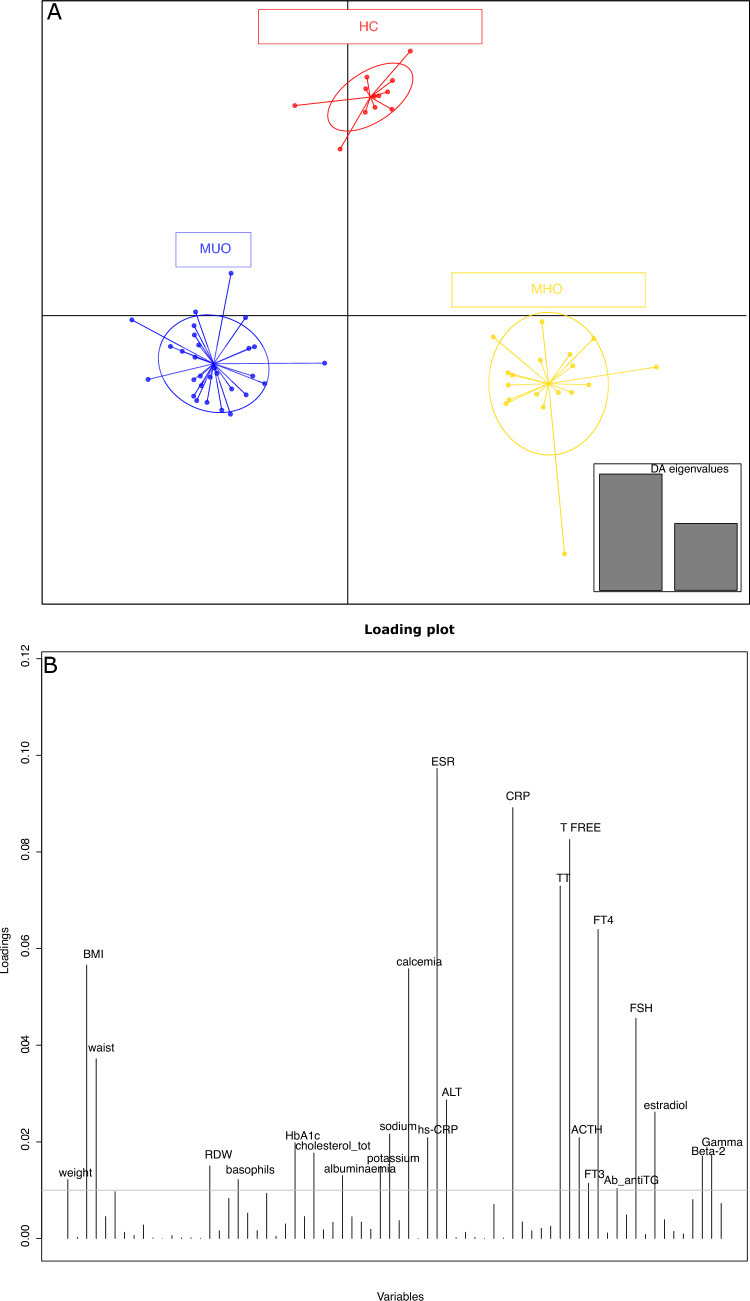
**A** DAPC based on the “a posterior” sample assignment. Based on two eigen-values, samples were assigned to clusters using their “a posterior” group belonging. **B** DAPC loadings. Most impacting variables higher than the arbitrary threshold (0.01)

A discriminant analysis of principal components (DAPC) was performed to understand if, based on variable loading, a sample stratification subsists without any group assignment. As emerged from the BIC curve (Supplementary Fig. S1) the better model provides for the separation of samples into three a priori clusters. When sample were “a posterior” assigned to groups, the three cohorts of subjects (HC, MHO, and MUO) plotted in three differentiated groups (Fig. 2A) and confirmed the a priori group stratification. The DAPC loading plot evidenced those variables (Fig. 2B) that had the greatest impact i.e., BMI, calcemia, erythrocyte sedimentation rate (ESR), CRP, total testosterone (TT), testosterone (T) free, thyroxine (FT4), and follicle stimulating hormone (FSH).

### Food questionnaires analysis

The nutritional values of volunteer’ diets have been estimated and results were reported in Supplementary Table S1. As expected, the whole average caloric intakes were similar in the three groups (P-value > 0.05).

Some statistically significant differences emerged for macronutrient and micronutrient values. In detail, two-group statistics elucidated how compared to HC volunteers, MUO and MHO diets reported greater amount of starch, edible part, total mineral salts, vitamin B1 (Thiamine), C18:1 oleic, and a lower amount of manganese. Moreover, compared to controls, MUO diets exhibited significantly lower quantities of sodium, potassium, iodine, and alpha-tocopherol, and higher cysteine.

By comparing diets from the two sub-cohorts, MUO showed higher quantities of cystine and C22:0 behenic acid, whereas MHO diet was characterized by greater quantities of tyrosine, vitamin B3 (niacin), and vitamin B6 (pyridoxine).

### Fecal volatilome

VOCs from fecal samples were analysed and quantified using HS-SPME GC–MS analyses. One hundred and seventeen volatile metabolites, ascribed to aldehyde, ester, ketone, indole, terpene, and alkane classes, were overall identified. Specifically, 112 volatiles were harboured by HC, 98 by the MHO group, and 96 by the MUO group. Eighty-nine compounds out of the total had the 100% of prevalence with respect to all the evaluated groups, whereas 7 of them (i.e., 2-methyl-1-butanol, trimethyl-pyrazine, propanoic acid butyl ester, butanoic acid-2-methylpropyl ester, dimethyl pentasulfide, acetic acid butyl ester, and 3-phenylpropanol) were absent in the two cohorts of obesity.

**Fig. 3:**
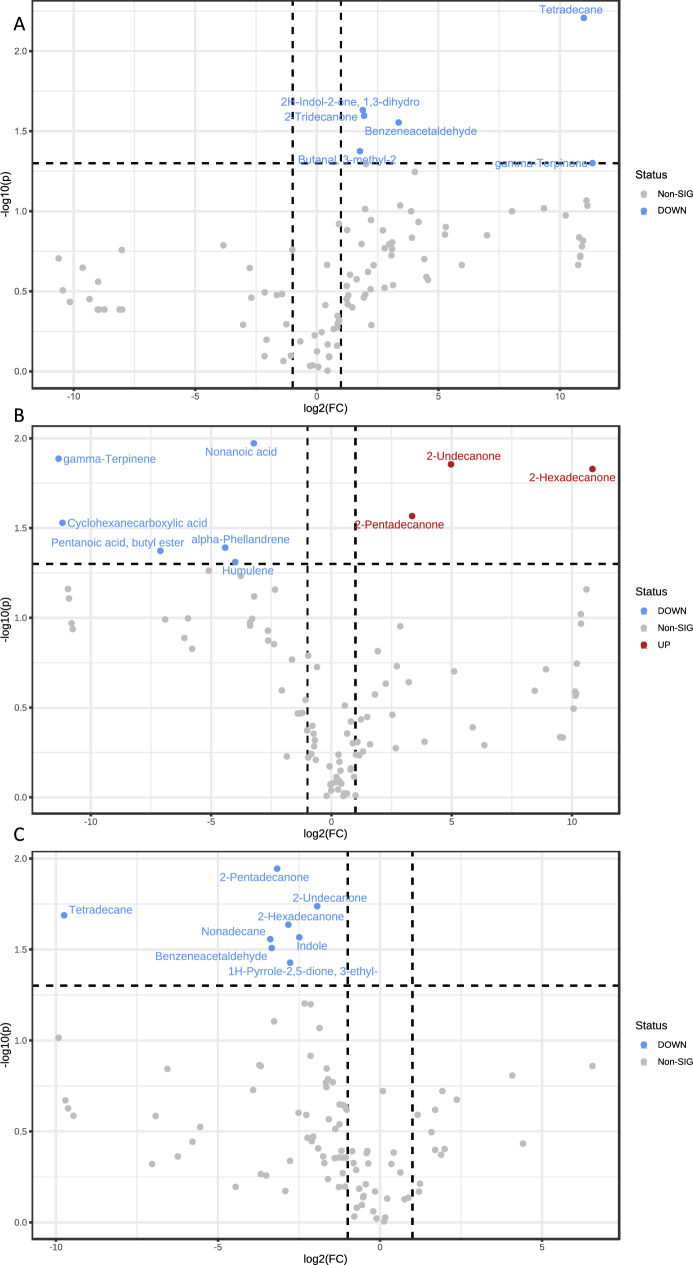
Statistically significant VOCs emerged from non-parametric Wilcoxon rank-sum test combined with fold change (FC) analysis. Because of the chosen comparison direction MHO/HC (panel (**A**)), MUO/HC (panel (**B**)), and MHO/MUO (panel (**C**)) increased and decreased metabolite concentrations in HC, MUO, and MHO groups have been marked as down (blue) and up (red) regulated, respectively. The -log10 (*p values*) is meaningful of the level of significance of each VOC and has been plotted versus the log2 fold change. It represents the difference between the levels of expression for each VOC between the two groups

A list of statistically significant VOCs emerged when groups were compared by meaning of non-parametric Wilcoxon rank-sum test combined with fold change (FC) analysis. Moreover, as shown in the volcano plot (Fig. 3A), the MHO group presented lower quantity of tetradecane, 2H-indol-2-one-1,3-dihydro, 2-tridecanone, benzeneacetaldehyde, butanal-3-methyl-2, and gamma terpinene versus HC. Compared to the healthy group, MUO subjects showed greater amount of nonanoic acid, gamma terpinene, cyclohexanecarboxylic acid, pentanoic acid, butyl ester, alpha phelladrene, and humulene and an up-regulation of 2-pentadecanone, 2-undecanone and 2-hexadecanone (Fig. 3B). Finally, lower levels of nonadecane, indole, 1H-pyrrole-2,5-dione, 3-ethyl-4-methyl, 2-pentadecanone, 2-undecanone, and 2-hexadecanone were observed in MHO as compared to MUO (Fig. 3C).

### SCFA quantification

A targeted GC–MS analyses of SCFAs was carried out. In detail, acetic, propanoic, butanoic, isobutyric, and isovaleric acids were analysed and (*p value* > 0.05) except for butanoic acid, whose concentrations resulted lower (*p value* < 0.05) in MUO subjects compared to MHO group, did not statistically differed (Supplementary Fig. S2).

### Real time PCR

**Fig. 4:**
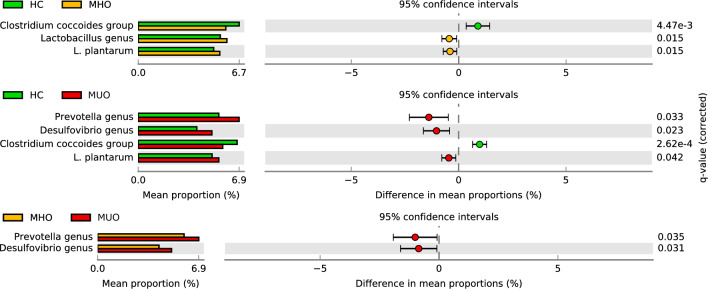
Statistically significant qPCR tested taxa emerging from pairwise comparison among healthy (HC), metabolically healthy obesity (MHO), and metabolically unhealthy obesity (MUO) sample cohorts. Groups were compared by applying a Benjamini–Hochberg corrected Welch’ test and corrected P-values (q-values) have been reported

To inspect the commensal taxa representative as much as possible of the gut microbiota, qPCR primers were selected for the identification of known genera, groups, and species harbouring the human gut. qPCR copy number log values have been reported in Supplementary Table S2. Few investigated taxa significantly differed between groups (Fig. 4).

In detail, *C. coccoides* group resulted to have lower CN log values in obesity groups than HC. Gut microbiota of MHO subjects presented increased amounts of *Lactobacillus* genus and *Lp. plantarum* than HC volunteers. Finally, MUO subjects showed a higher abundance of *Prevotella* and *Desulfovibrio* genera, compared to both HC and MHO subjects, and higher *Lp. plantarum* CN log compared to HC group.

## Discussion

This observational clinical study relies on the power of statistical multivariate approaches useful in reducing low-impacting variable noise, and in selecting the most contributing variables that discriminated MHO, MUO and HC cohorts. In addition, for the first time, groups of obese patients and healthy subjects followed a restrictive caloric diet in the range 800 < x < 1200 kcal per day. This allowed us to lower the impact of diet regimens on considered sample stratifications. Downstream to all the inspected statistical results, specific markers of obesity-related comorbidities, i.e. inflammation, liver damage, immunity response, and hormone metabolism profiled MUO patients Although some significant variables including BMI and weight were shared between MHO and MUO, the metabolic profile confirmed the well-known differences between the two obese phenotypes. Specifically, higher levels of HbA1c, HOMA-IR, total cholesterol, triglycerides, AST, ALT, and FBG, as well as lower GFR and albumin levels were detected in MUO, corroborating the association of visceral adiposity with reduced insulin sensitiveness, metabolic alterations, and low-grade inflammation. Lower GFR and hypoalbuminemia are also known to correlate with morbid obesity, thus increasing the risk of chronic kidney disease onset [12]. In addition, higher levels in AST and ALT enzymes observed in MUO as compared to HC and MHO, describe the presence of liver disease and the progression of hepatic dysfunction [13].

The worsening of renal conditions is a clear indication of inflammation, as stated for patients suffering from pathological obesity, that are characterized by impaired insulin sensitivity and higher levels of HbA1c too [14], a diagnostic marker of pre-diabetes and T2D diabetes.

Moreover, HOMA-IR resulted to be higher in MUO compared to MHO patients, and our rotating factor analysis included it as part of the second high-loading factor (greater than 0.7).

MUO reported lower level of albumin compared to MHO. Reduced albumin serum levels have been weighted as marker of alteration in energy intake metabolism and liver damage. In addition, we found the presence of dyslipidaemia (increased triglycerides and cholesterol) in MUO as compared to HC.

Other inflammatory markers, part of the first rotating analysis factor, associated with systemic inflammation in patients with obesity i.e., CRP, hsCRP, and ESR, were increased in MUO versus HC. In these pathologic conditions, further evidence is prompted by the white blood cells and platelets that significantly increased compared to HC. Cytokines derived by inflammation, along with adipokines, such as leptin, induce leucocytosis together with a higher pituitary secretion of TSH. This hormone impacts adiposity, adipogenesis regulation, appetite, insulin resistance, and weight gain [15].

Furthermore, as expected, we also detected higher levels of insulin in MUO patients compared to HC, associated with obesity and inflammation [16].

Finally, levels of 25-OH vitamin D, an anti-inflammatory and anti-adipogenic factor, were significantly reduced in MUO compared to HC group. Vitamin D is a fat-soluble mediator mainly stored in adipose tissue and its circulating reduction in obesity could reflect a volumetric dilution in fat compartment [17]. Noteworthy, the 25-OH vitamin D levels of MHO patients showed no statistically significant differences as compared to HC subjects. This indicates a different distribution of adipose tissue in two obese phenotypes that could differently impact on the factor release.

Although the caloric range in the three groups was equivalent, some statistically significant differences emerged for specific nutrients. Both groups of obesity showed a higher consumption of starch and manganese, but lower intake of total mineral salts, thiamine, and oleic acid when compared to HC group. Similar results have been obtained by previous studies where an excessive blood level of manganese resulted positively associated with increased visceral adipose tissue mass [18], as well as low consumption of MUFA resulted associated with a higher risk of obesity and metabolic consequences [19].

Interestingly, MUO individuals showed differences in the consumption of some micronutrients: a lower intake of iodine, known to enhance thyroid problems [20], together with reduced levels of alpha-tocopherol, a vitamin able to favour glycaemic control, and inversely related to BMI in morbid obesity [21]. Conversely, higher intake of cystine, an amino acid with adipogenic property and frequently related with fat accumulation [22], was observed in MUO as compared to HC. Interestingly, cystine intake resulted higher in MUO also in comparison with MHO subjects, suggesting that this amino acid could differentiate these two different obesity phenotypes. A comparable intake of pyridoxine, vitamin with positive metabolic effectiveness [23], and niacin, with a potential role in improving cardiovascular risk and dyslipidaemia [24], was observed between MHO and HC, but not in MUO individuals, thus suggesting that these micronutrients could prevent the metabolic worsening of MHO towards a more severe obesity phenotype.

By targeting the intestinal microbial population qPCR analysis showed that MHO individuals were characterized by higher abundance of *Lactobacillus* genus compared to HC. The microbiota of both MUO and MHO showed higher levels of *Lactiplantibacillus plantarum* versus HC. Although species included in *Lactobacillus* genus exert beneficial effects and anti-obesity properties, studies revealed that some species were associated with obesity [25]. Armougom et al. [26] reported a correlation between the concentration of *Lactobacillus* spp. and the weight gain in patients with obesity, compared to healthy and anorexic subjects.

In addition, MUO were characterized by higher quantities of *Prevotella* and *Desulfovibrio* genera when compared with MHO and HC. Previous studies showed high prevalence of *Prevotella* genus in patients with obesity and observed differences in terms of abundances in both the metabolic obesity classes [27]. Recent literature findings reported how subjects who underwent a restrictive Mediterranean diet exhibit a decrease in *Prevotella copri* species with a concomitant improvement in insulin resistance [28] and, at the same time, indicated how an increase in Prevotella/Bacteroidetes ratio reflects a metabolic shift that included important changes in carbohydrate metabolism [29, 30].

Noteworthy, the inspection of fiber enriched diet effects, including a reduction in BMI and waist circumference levels, revealed a strong correlation with *Prevotella* genus increased abundances [31–33].

This action on weight reduction would be dependent on the higher levels of SCFAs, in particular propionate [31], with an enhanced activity of *Prevotella* in the liver. Moreover, this taxa is active in reducing serum cholesterol and hepatic lipogenesis, acting on anti-inflammatory pathways, thus preventing weight gain in human and rodents [34]. Thus the restricted caloric regimen in our cohort definitely led *Prevotella* to increase in MUO compared to MHO and HC sample sets.

In a rat model, *Prevotella* appeared to regulate feeding behaviour since its abundance was positively associated with appetite-regulating hormones as ghrelin, known to stimulate the sense of hungry, and, was negatively correlated with leptin, the anorectic hormone [35]. Furthermore, as discussed above, leptin promotes the differentiation of granulocytes, inducing leucocytosis, the well-known process which accompain the low-grade of inflammation of morbid obesity [36]. On the other hand, *Desulfovibrio* is positively correlated with different dysmetabolic conditions [37]. Furthermore, this genus plays a role in the development of NAFLD where it could lead to increase the intestinal permeability and the upregulation of the gene involved in fat storage of liver cells [38].

Of note, lower concentrations of *Clostridium coccoides group* were observed in obese sets as compared to HC. The controversial role of *C. coccoides* is still under debate; some studies reported a positive correlation with the worsening of obesity [39], while others discussed the lower abundance in mice exposed to high-fat diet [40]. Furthermore, over the negative correlation with insulin level and HOMA-IR, this taxon was found to be negatively associated with the ghrelin level [7]. Some evidence also support the beneficial role of this taxon as a butyrate-producer [41]. In fact, a reduction of *C. coccoides group* observed in MUO was accompanied by the decrease of fecal amount of butanoic acid versus MHO. Butanoic acid impacts the i) increase of leptin release and insulin sensitivity, ii) decrease of hypercholesterolemia and hepatic steatosis, iii) decrease of HOMA-IR index and iv) modulation of cellular events that regulate the assembly and delivery of lipoproteins.

Based on fecal volatilome profiles, MUO subjects reported significant lower concentration of 2H-indol-2-one-1,3-dihydro and nonanoic acid as compared to HC. Nonanoic acid is associated with beneficial effects and antimicrobial properties [42]. 2H-indol-2-one-1,3-dihydro has been described to activate the aryl hydrocarbon receptor [43], whose activation prevents cytokine induction and defends gut barrier integrity against damage in obesity [44]. Indole, a compound associated to major adverse cardiovascular events [45] resulted higher in MUO compared to MHO group.

Furthermore, compared to HC, MUO showed lower quantities of humulene, gamma-terpinene, and alpha-phellandrene, present in herbs with antioxidant, anti-inflammatory, and anti-obesity properties [46].

MUO group reported higher concentration of hexadecanoic acid, a derivative of 2-hexadecanone, known to correlate with insulin resistance and T2D, by increasing the synthesis of deleterious lipids and inflammatory mediators [47]. Similarly, 2-undecanone, an healthy metabolite able to mediate anti-inflammatory and antioxidant effects whose production is ascribed to *L. plantarum* [48] increase in MUO. This ketone inhibits some proteins and cytokines related to nuclear factor-kB (NF-kB) pathway involved in the antioxidant response [49]. Therefore, the greater amounts of 2-undecanone and *L. plantarum* observed in MUO individuals probably could be related to the inflammatory milieu associated with morbid obesity. Among ketone compound class, 2-tetradecanone was less represented in fecal samples of MHO patients as compared to controls. Some evidence reported antioxidant, anti-inflammatory, and anti-diabetic activities of its ester derivative ethyl tridecanoate [50]. In addition, MUO group as compared to HC showed lower levels of cyclohexanecarboxylic acid whose derivative, the 4-phenylpiperidine-1-carbonyl cyclohexanecarboxylic acid is known to inhibit the diacylglycerol acyltransferase 1, an enzyme involved in triglyceride biosynthesis [51]. Finally, a reduction of pentanoic acid butyl ester amount was observed in MUO than HC. This compound belongs to fatty acid ester derivative class, which reported beneficial effects against obesity, by regulating serum lipid profiles and by inducing adipocyte apoptosis [52].

Although preliminary, our investigation approach has some limitations. The here applied analytical strategy, based on caloric intake data gathered as three-day food questionnaires, prevents the introduction of biases related to non-adherent subjects. Dealing with the number of samples, due to its explanatory nature, this pilot investigation targeting MUO and MHO obese patients does not rely on a statistical power calculation.

Undoubtedly, the reported real time PCR findings would take advantage from a metataxonomics sequencing analysis that would crucially strengthen the connection between VOCs and fecal taxa.

## Conclusions

In our observational study, we analysed for the first time the significant differences in clinical biochemical parameters, selected intestinal microbial taxa, and volatilome between subjects with metabolically healthy and unhealthy obesity, compared to healthy control volunteers with a daily calorie intake set in a specific range. Based on a strong statistical rationale, all conducted analyses made us confident in ascertaining a MUO specific clinical signature, highlighting how metabolically healthy obesity most likely represents a transient obesity phenotype. These data could represent as a prognostic marker tool useful in monitoring the obesity-associated metabolic comorbidities. Future studies are needed to understand the molecular mechanisms beyond the intestinal dysbiosis in the development and progression of obesity.

## Supplementary Information

Below is the link to the electronic supplementary material.

Supplementary file1 (DOCX 33 KB)

Supplementary file2 (PDF 26 KB)

Supplementary file3 (PDF 54 KB)

Supplementary file4 (DOCX 165 KB)

## Data Availability

The datasets used and/or analysed during the current study available from the corresponding author on reasonable request.
