# Supplementary material for: Gut microbiota and fecal volatilome profile inspection in metabolically healthy and unhealthy obesity phenotypes
Source: J Endocrinol Invest. 2024 Jun 21;47(12):3077–90. doi: 10.1007/s40618-024-02379-2 (PMC11549234; doi:10.1007/s40618-024-02379-2)
Supplement: Supplementary file 1 — Supplementary file1 (DOCX 33 KB) [file 40618_2024_2379_MOESM1_ESM.docx]

**Online supporting Information**

**Fecal metabolome analysis**

The VOCs analysis was performed with a Clarus 680 (Perkin Elmer, Beaconsfield, UK) gas chromatograph equipped with an Rtx-WAX column (30 m x 0.25 mm i.d., 0.25 µm film thickness) (Restek), and coupled to a Clarus SQ8MS (Perkin Elmer). The HD-SPME extraction method with DVB/CAR/PDMS (Supelco, Bellefonte, PA, USA) fiber and GC-MS analyses were performed according to Portincasa et al. (1). Each chromatogram was analysed for peak identification using the National Institute of Standard and Technology 2008 (NIST) library. A peak area threshold of > 1,000,000 and 85 % or greater probability of match was used for VOC identification, followed by manual visual inspection of the fragment patterns when required. 4-methyl-2pentanol (final concentration 1 mg/L) was used as internal standard (IS). The final concentration was obtained by interpolation of the relative areas versus IS area and expressed in µg/g of IS.

**Short Chain Fatty Acids analysis and quantification**

Standard curves were constructed with pure standards of acetic, butyric, propionic, isobutyric, and isovaleric acid (Sigma-Aldrich). 4-methyl-2-pentanol (final concentration of 1 mg/L) was used as IS. Total Ion Current (TIC) mode was used to obtain typical ions with a special mass-to-charge ratio relative to each SCFA. The Selective Ion Monitoring (SIM) mode was then used to evaluate the concentration of each compound (2). SCFA relative peaks in fecal samples were integrated and their absolute concentrations (µg/g) were calculated by using the calibration curve equation.

**DNA Extraction and Real Time PCR**

An aliquot (500 µL) of each stool samples was used for total DNA extraction. Samples were diluted in 1 mL of PBS-EDTA (phosphate buffer 0.01 M, pH 7.2, 0.01 M EDTA) and centrifuged (14,000 × g at 4 °C) for 5 min. Pellet was washed twice to reduce the presence of PCR inhibitors. The extraction was performed using the FastDNA® Pro Soil-Direct Kit (MP Biomedicals, CA., USA). The quality check of the final DNA was carried out by spectrophotometric measurement at 260, 280, and 230 nm with the use of NanoDrop® ND-1000 Spectrophotometer (ThermoFisher Scientific Inc., MI., Italy).

Based on several evidence reporting the consortium bacteria composition of the intestinal microbiota (3-6), we selected and quantified through Real Time PCR (qPCR) the contribution of a subset composed of bacterial genera, groups, and species that reflects the intestinal environment and that plays a role in the pathogenesis of dysmetabolism associated with obesity. Primers used for microbial investigation have been listed and described in Online Supporting Information Table 1. qPCR reactions were carried out by the Applied Biosystems 7300 Real-Time PCR System (ThermoFisher Scientific Inc., MI., Italy). The total reaction mix (25 μl) contained 12.5 μl of SYBR Green Mix (# 1725271, Bio-Rad Laboratories S.r.l., Milano, Italy), 0.1 μl of 0.2 μM of primer, 11.4 μl of DNase and RNase-free water, and 1 μl of DNA template (40 ng). Each reaction was performed in duplicate. The amplification program consisted of 1 cycle of 95 °C for 2 min, followed by 40 cycles of 95 °C for 5 s, appropriate annealing temperature (Online Supporting Information Table 1) for 30s and 72 °C for 35 seconds.

For each primer a standard curve was previously constructed by using serial dilutions of pure culture extracted DNA. The qPCR results (cycle threshold, C_T_) were converted in Copy Number (CN). The CN was calculated based on DNA concentration and amplicon length. The standard curves were obtained by C_T_ and CN(Log) interpolation.

**Online Supporting Information Table 1.** List of primer and relative qPCR conditions.

| *Genus/species* | *Primers* | *Primer sequence (5' - 3')* | *Gene* | *Product size (bp)* | *T annealing (°C)* | *Reference* |
| --- | --- | --- | --- | --- | --- | --- |
| *Lactobacillus* | Lac-F | AGCAGTAGGGAATCTTCCA | *16S rRNA* | 341 | 58 | (3) |
|  | Lac-R | CACCGCTACACATGGAG |  |  |  |  |
| *Bifidobacterium* | Bifid-F | CTCCTGGAAACGGGTGG | *16S rRNA* | 550 | 55 | (3) |
|  | Bifid-R | GGTGTTCTTCCCGATATCTACA |  |  |  |  |
| *Prevotella* | g-Prevo-F | CACRGTAAACGATGGATGCC | *16S rRNA* | 528 | 55 | (3) |
|  | g-Prevo-R | GGTCGGGTTGCAGACC |  |  |  |  |
| *Atopobium* cluster | Atopo-F | GGGTTGAGAGACCGACC | *16S rRNA* | 190 | 55 | (3) |
|  | Atopo-R | CGGRGCTTCTTCTGCAGG |  |  |  |  |
| *Desuldovibrio* | Dsv-F | CCGTAGATATCTGGAGGAACATCAG | *16S rRNA* | 135 | 63 | (3) |
|  | Dsv-R | ACATCTAGCATCCATCGTTTACAGC |  |  |  |  |
| *Bacteroides fragilis* group | Bfra-F | ATAGCCTTTCGAAAGRAAGAT | *16S rRNA* | 495 | 50 | (3) |
|  | Bfra-R | CCAGTATCAACTGCAATTTTA |  |  |  |  |
| *Clostridium coccoides* group | Ccoc-F | AAATGACGGTACCTGACTAA | *16S rRNA* | 440 | 50 | (3) |
|  | Ccoc-R | CTTTGAGTTTCATTCTTGCGAA |  |  |  |  |
| *Clostridium leptum* group | sg-Clept-F | GCACAAGCGTGGAGT | *16S rRNA* | 239 | 55 | (3) |
|  | sg-Clept-R3 | CTTCCTCCGTTTTGTCAA |  |  |  |  |
| *Lactiplantibacillus plantarum* | Lp-F | AAAATCATGCGTGCGGGTAC | *pyrG* | 261 | 55 | (4) |
|  | Lp-R | ATGTTGCGTTGGCTTCGTCT |  |  |  |  |
| *Lacticaseibacillus rhamnosus* | LrhamF | GGACAGGTAGAAAGTCAAACGA | *mutL* | 186 | 65 | (4) |
|  | LrhamR | GCTGACCGTAAACGCAATCTTAG |  |  |  |  |
| *Limosilactobacillus reuteri* | Lreu-1 | CAGACAATCTTTGATTGTTTAG | *16S-23S spacer region* | 305 | 60 | (4) |
|  | Lreu-4 | GCTTGTTGGTTTGGGCTCTTC |  |  |  |  |
| *Limosilactobacillus fermentum* | Lfer-3 | ACTAACTTGACTGATCTACGA | *16S-23S spacer region* | 191 | 50 | (4) |
|  | Lfer-4 | TTCACTGCTCAAGTAATCATC |  |  |  |  |
| *Bifidobacterium longum* | BiLON-1 | TTCCAGTTGATCGCATGGTC | *16S rDNA* | 831 | 55 | (5) |
|  | BiLON-2 | GGGAAGCCGTATCTCTACGA |  |  |  |  |
| *Bifidobacterium breve* | BiBRE-1 | CCGGATGCTCCATCACAC | *16S rDNA* | 288 | 55 | (5) |
|  | BiBRE-2 | ACAAAGTGCCTTGCTCCCT |  |  |  |  |
| *Bifidobacterium infantis* | BiINF-1 | TTCCAGTTGATCGCATGGTC | *16S rDNA* | 828 | 55 | (5) |
|  | BiINF-2 | GGAAACCCCATCTCTGGGAT |  |  |  |  |
| *Bifidobacterium bifidum* | BiBIF-1 | CCACATGATCGCATGTGATTG | *16S rDNA* | 278 | 55 | (5) |
|  | BiBIF-2 | CCGAAGGCTTGCTCCCAAA |  |  |  |  |
| *Bifidobacterium adolescentis* | BiADO-1 | CTCCTGGAAACGGGTGG | *16S rDNA* | 279 | 55 | (5) |
|  | BiADO-2 | GGTGTTCTTCCCGATATCTACA |  |  |  |  |
| *Akkermansia muciniphila* | AmucF | CAGCACGTGAAGGTGGGGAC | *16S rDNA* | 329 | 60 | (6) |
|  | AmucR | CCTTGCGGTTGGCTTCAGAT |  |  |  |  |

**Statistical analyses**

With the aim of identifying statistically significant differences in individual analyses and to correlate the results of the study, we adopted stringent statistical analyses to find out significant results.

Focusing on clinical data, an exploratory factor analysis was performed by using the principal-component factor model to reduce the number of variables. Specifically, the factor analysis was run based on the principal-component factor model which allowed for the consideration of unique variables with a higher loading. The statistical principle on which this analysis is based stand upon the linear combination of variables that contain most of the information.

To restrict the entire panel to few factors with a strong clinical interpretation, we *a priori* picked eigenvalues equal to or greater than two. A graphical assessment of the eigenvalues was performed. As post-estimation tools we used the orthogonal rotation (varimax) to give more weight to large initial loadings. Finally, the Kaiser–Meyer–Olkin (KMO) measure of sampling adequacy was applied.

Two-group comparisons were performed through the application of a Welch’ T-test corrected (Benjamini-Hochberg) test or by using the non-parametric Wilcoxon rank-sum test combined with fold change (FC) analysis graphically rendered as volcano plots.

The clustering among our samples was inspected by means of the discriminant analysis of principal components (DAPC), a multivariate analysis based on the selection of few synthetic variables (linear combinations of the original ones). Specifically, in a first step, the DAPC was run on the taxa abundance matrix without superimposing any metadata grouping condition and using the ‘find.clusters’ clustering algorithm. Subsequently, according to the obtained metadata information, the assigned dietary/physical activity intervention group of each sample was set as the a priori condition. The prior and posterior membership probabilities were computed and graphically translated using the ‘assignplot’ function within the “adegenet” R package v2.1.1 (https://cran.r-project.org/web/packages/adegenet/index.html (accessed on 20 June 2023). This allowed us to calculate the proportions of successful reassignments. To assess the weight of each variable, the taxa contributing most strongly to cluster separation were computed and plotted using the ‘assignplot’ function within the R adegenet package.

**References**

1. Portincasa P, Celano G, Serale N, *et al.* Clinical and Metabolomic Effects of Lactiplantibacillus plantarum and Pediococcus acidilactici in Fructose Intolerant Patients. *Nutrients* 2022;14:2488.

2. Hsu Y-L, Chen C-C, Lin Y-T, *et al.* Evaluation and Optimization of Sample Handling Methods for Quantification of Short-Chain Fatty Acids in Human Fecal Samples by GC-MS. *J Proteome Res* 2019;18:1948–1957.

3. Kwok L, Zhang J, Guo Z, *et al.* Characterization of fecal microbiota across seven Chinese ethnic groups by quantitative polymerase chain reaction. *PLoS One* 2014;9:e93631.

4. Jomehzadeh N, Javaherizadeh H, Amin M, Rashno M, Teimoori A. Quantification of Intestinal Lactobacillus Species in Children with Functional Constipation by Quantitative Real-Time PCR. *Clin Exp Gastroenterol* 2020;13:141–150.

5. Matsuki T, Watanabe K, Fujimoto J, *et al.* Quantitative PCR with 16S rRNA-gene-targeted species-specific primers for analysis of human intestinal bifidobacteria. *Appl Environ Microbiol* 2004;70:167–173.

6. Keshavarz Azizi Raftar S, Hoseini Tavassol Z, Amiri M, *et al.* Assessment of fecal Akkermansia muciniphila in patients with osteoporosis and osteopenia: a pilot study. *J Diabetes Metab Disord* 2021;20:279–284.
