# Supplementary material for: Gut microbiota and fecal volatilome profile inspection in metabolically healthy and unhealthy obesity phenotypes
Source: J Endocrinol Invest. 2024 Jun 21;47(12):3077–90. doi: 10.1007/s40618-024-02379-2 (PMC11549234; doi:10.1007/s40618-024-02379-2)
Supplement: Supplementary file 2 — Supplementary file2 (PDF 26 KB) [file 40618_2024_2379_MOESM2_ESM.pdf]

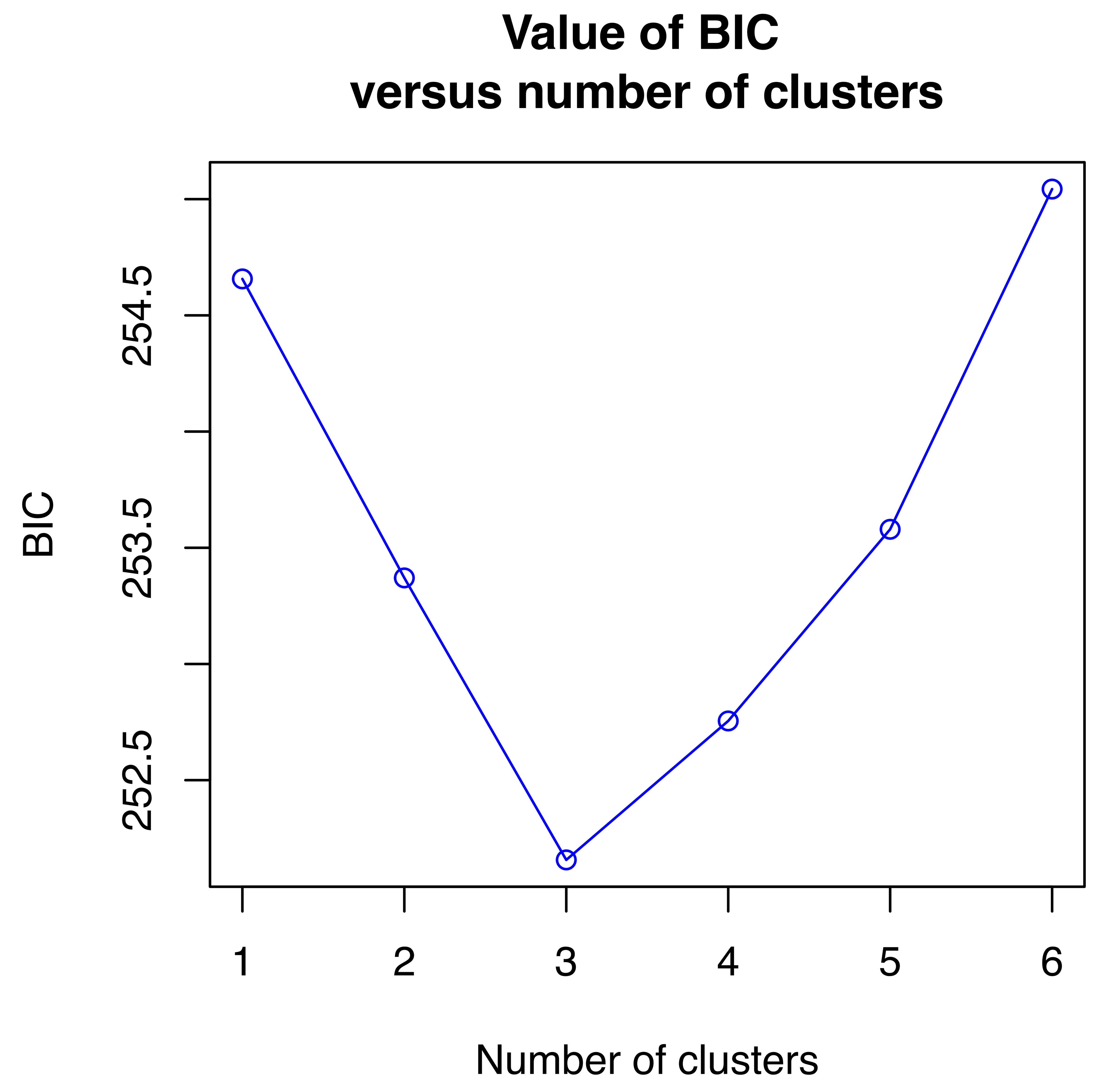
Value of BIC
versus number of clusters
BIC
252.5
253.5
254.5
1
2
3
4
5
6
Number of clusters

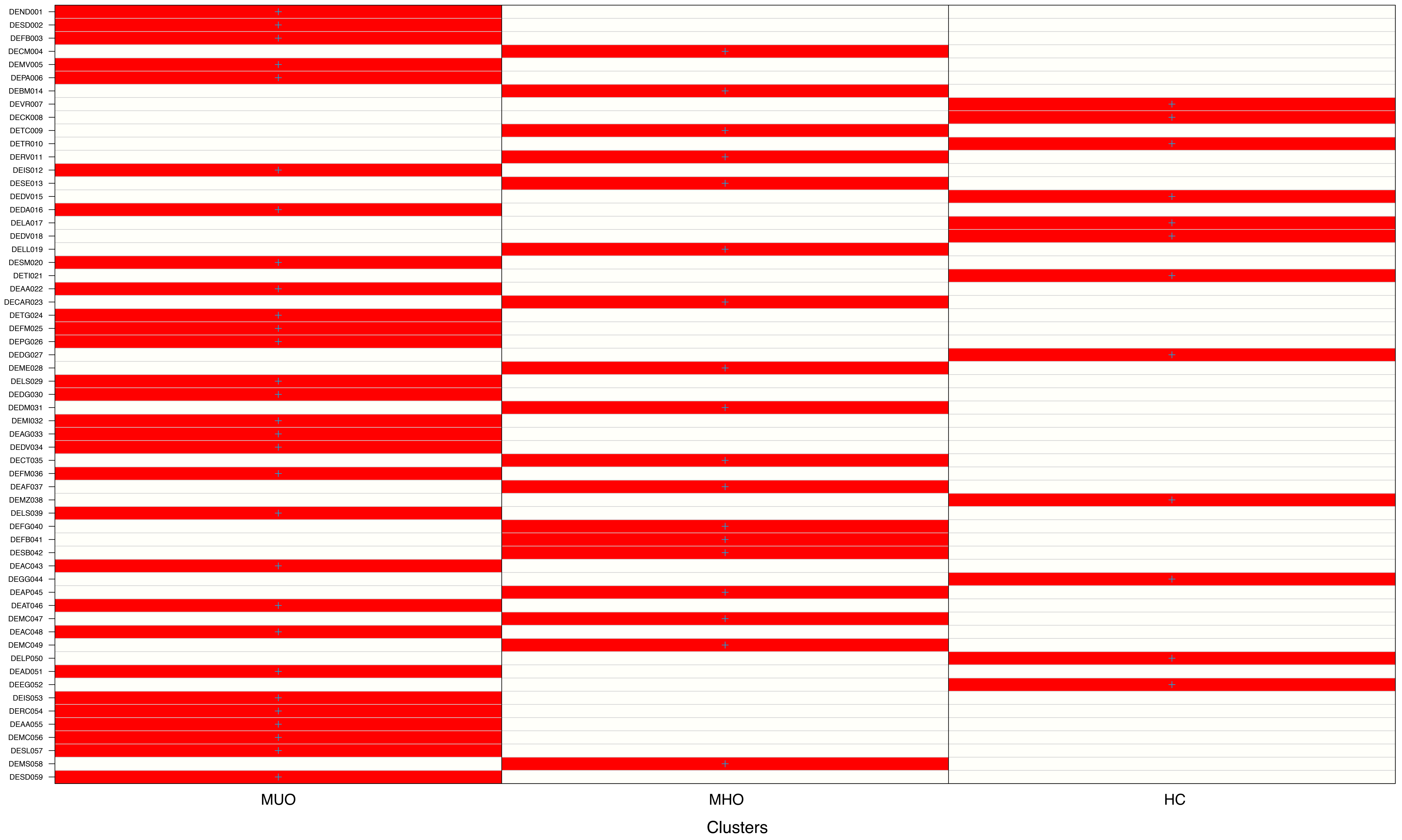
DEND001
DESD002
DEFB003
DECM004
DEMV005
DEPA006
DEBM014
DEVR007
DECK008
DETC009
DETR010
DERV011
DEIS012
DESE013
DEDV015
DEDA016
DELA017
DEDV018
DELL019
DESM020
DETI021
DEAA022
DECAR023
DETG024
DEFM025
DEPG026
DEDG027
DEME028
DELS029
DEDG030
DEDM031
DEMI032
DEAG033
DEDV034
DECT035
DEFM036
DEAF037
DEMZ038
DELS039
DEFG040
DEFB041
DESB042
DEAC043
DEGG044
DEAP045
DEAT046
DEMC047
DEAC048
DEMC049
DELP050
DEAD051
DEEG052
DEIS053
DERC054
DEAA055
DEMC056
DESL057
DEMS058
DESD059
MUO
MHO
HC
Clusters
