# Supplementary figures and images for: Gut microbiota and fecal volatilome profile inspection in metabolically healthy and unhealthy obesity phenotypes
Source: J Endocrinol Invest. 2024 Jun 21;47(12):3077–90. doi: 10.1007/s40618-024-02379-2 (PMC11549234; doi:10.1007/s40618-024-02379-2)

**Acetic Acid (C:2)**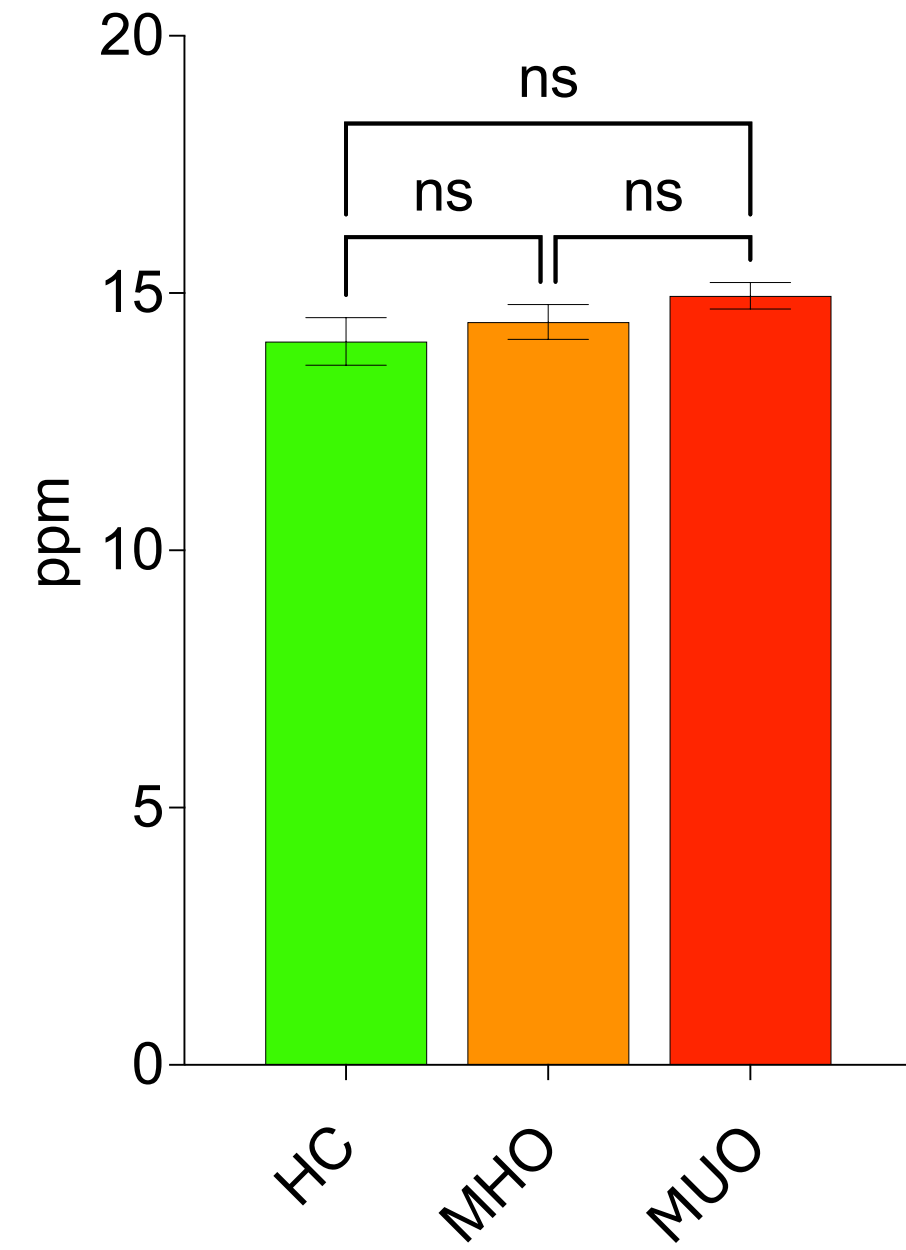**Propanoic Acid (C:3)**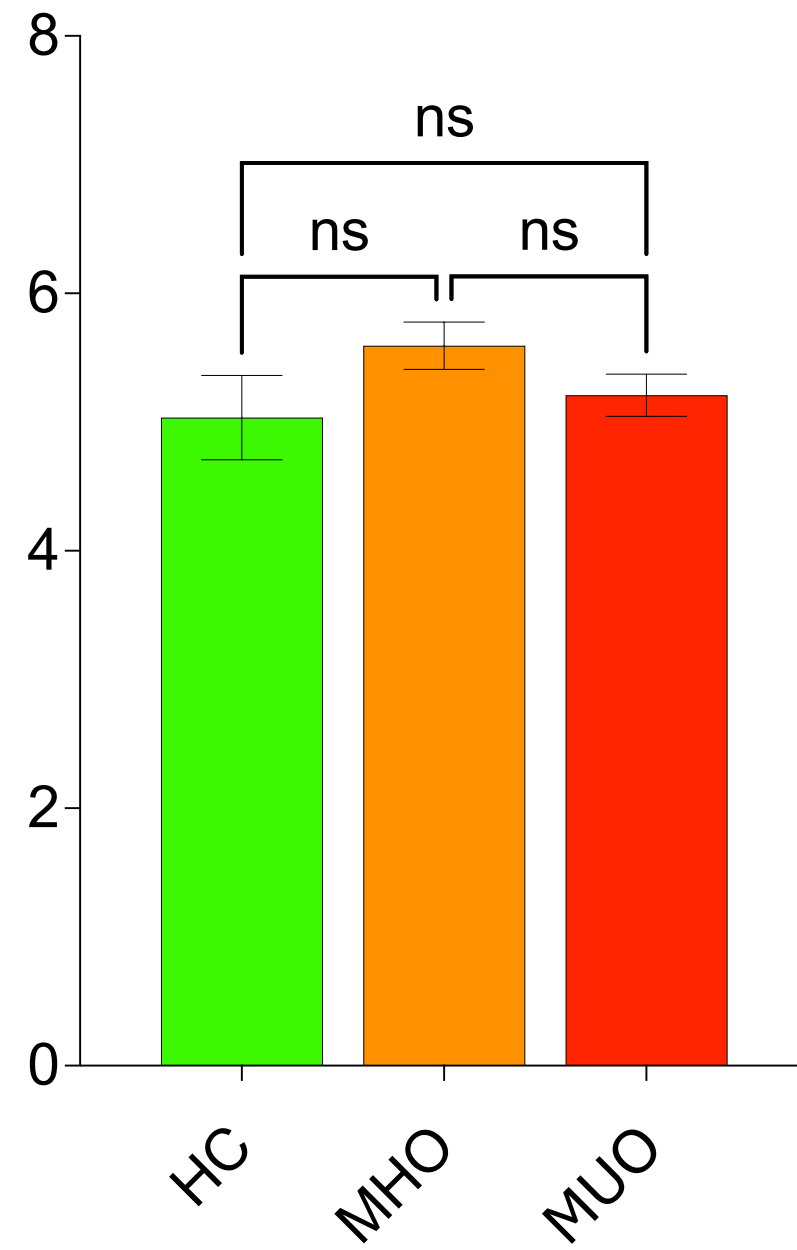**Butanoic acid (C:4)**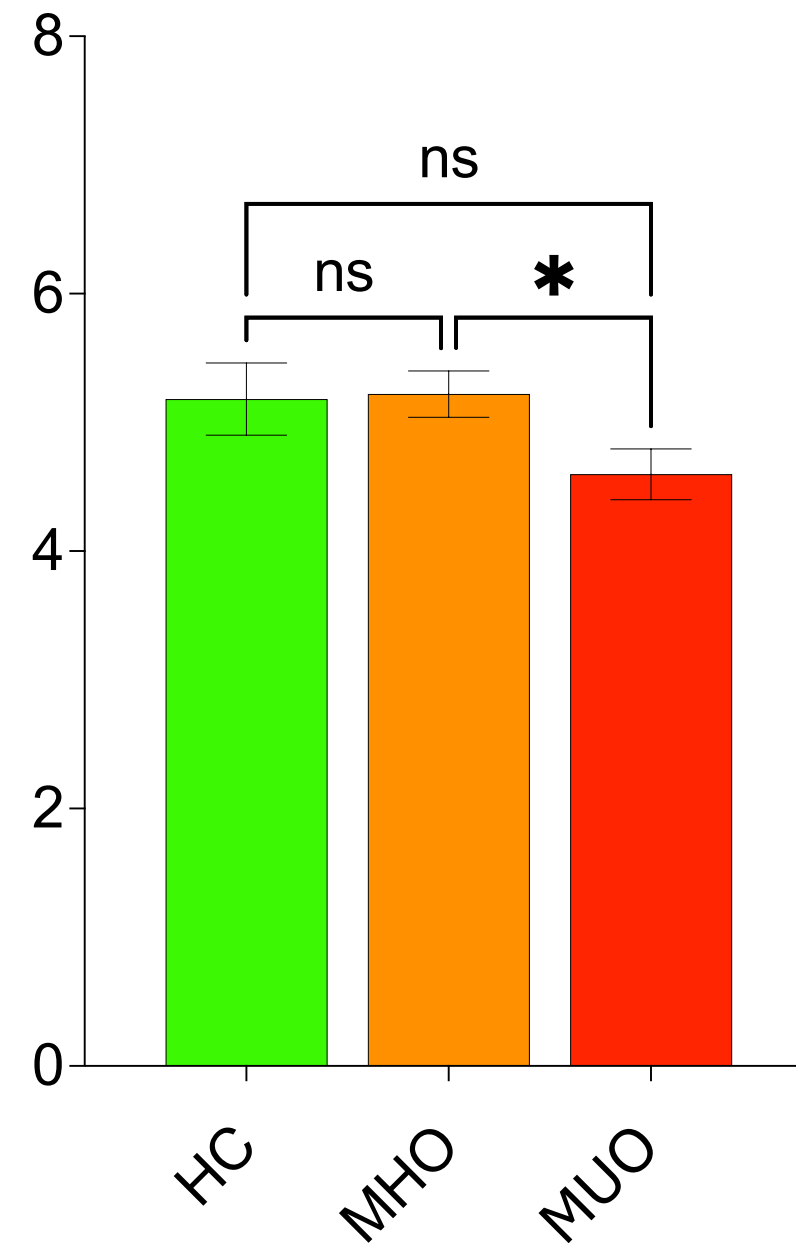**Isobutyric acid (C:4)**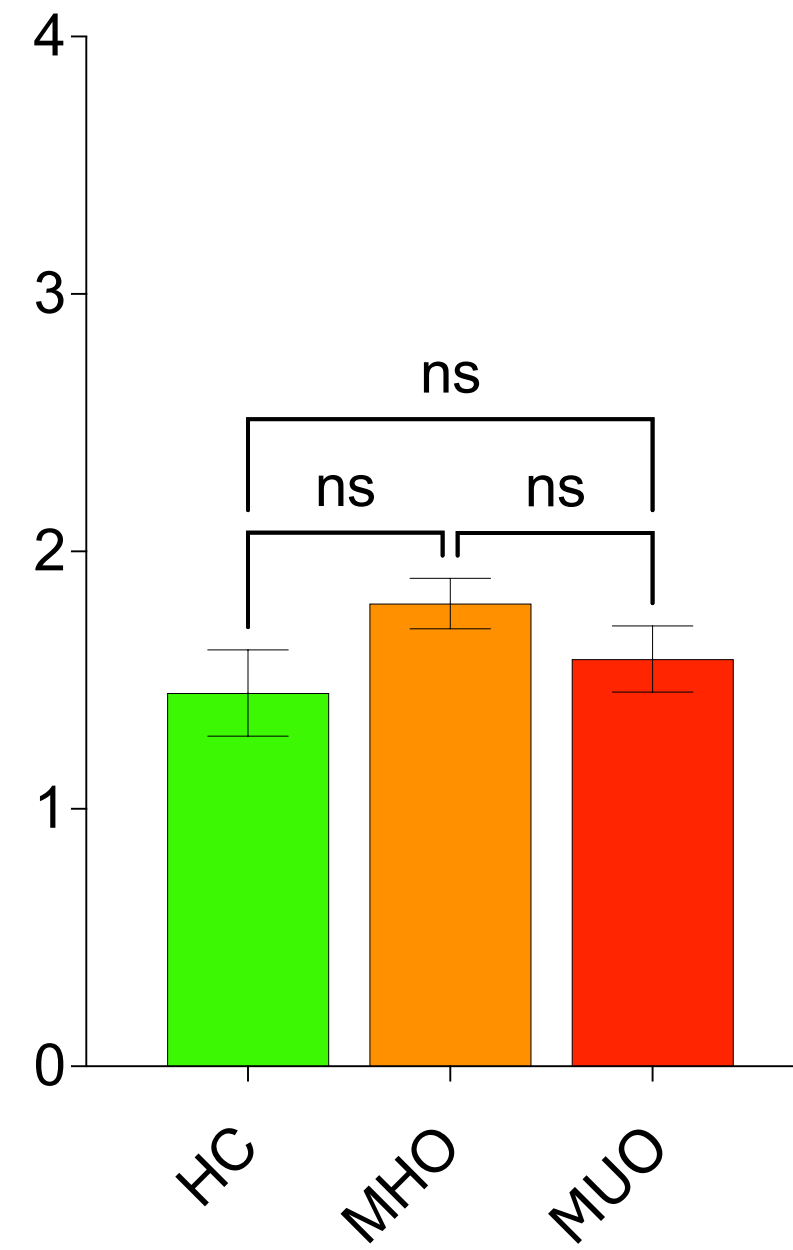**Isovaleric acid (C:5)**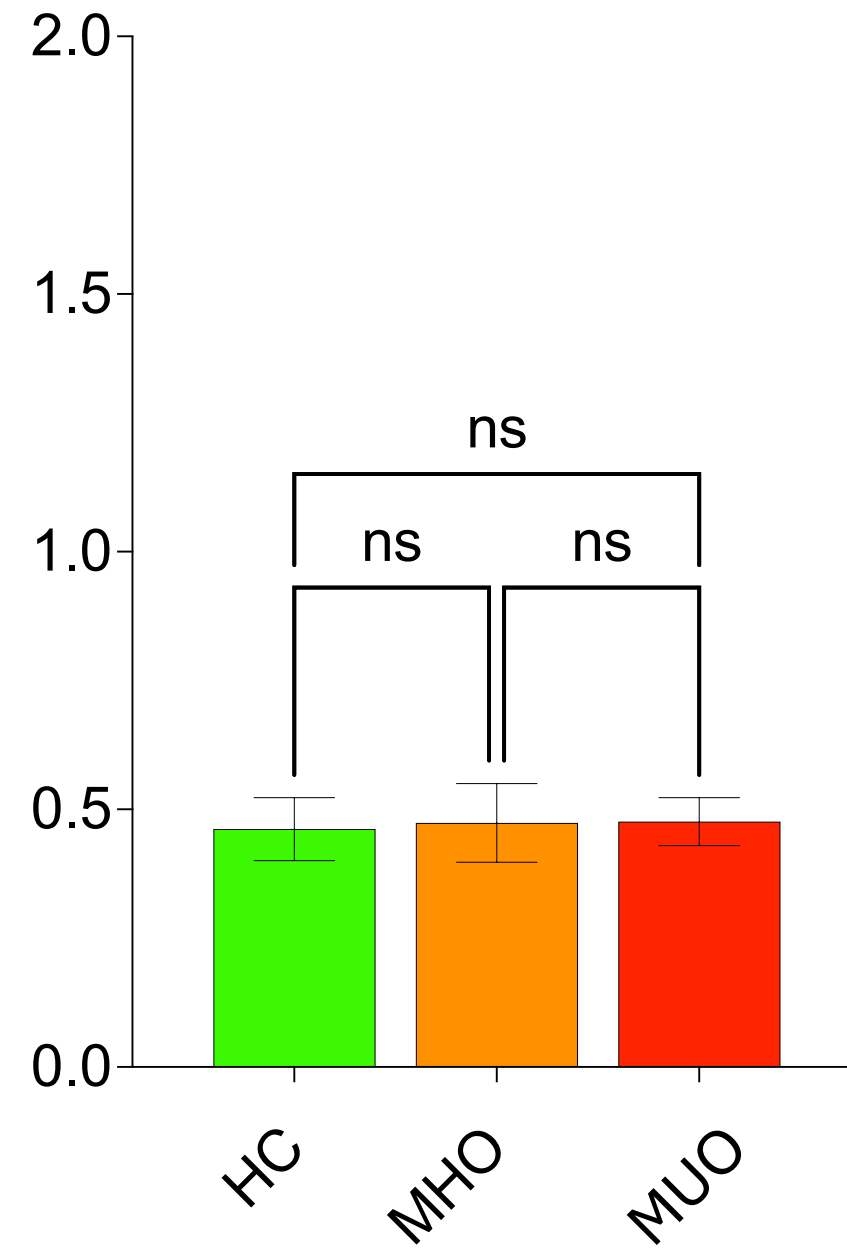

Supplement: Supplementary file 3 — Supplementary file3 (PDF 54 KB) [file 40618_2024_2379_MOESM3_ESM.pdf]
