# Supplementary material for: Gut microbiota and fecal volatilome profile inspection in metabolically healthy and unhealthy obesity phenotypes
Source: J Endocrinol Invest. 2024 Jun 21;47(12):3077–90. doi: 10.1007/s40618-024-02379-2 (PMC11549234; doi:10.1007/s40618-024-02379-2)
Supplement: Supplementary file 4 — Supplementary file4 (DOCX 165 KB) [file 40618_2024_2379_MOESM4_ESM.docx]

**Supplementary Table S1.** Daily nutritional values measured in the three groups of subjects (HC, MHO, and MUO). In the two group comparisons, q-values were reported only if the variable resulted to be statistically significant after correction by multiple test.

|  | Unit | HC | MHO | MUO | MHO  vs HC  *q value* | MUO  vs HC  *q value* | MUO vs MHO  *q value* |
| --- | --- | --- | --- | --- | --- | --- | --- |
| Macronutrients | | | | | | | |
| Kalories | Kcal | 1110.65 | 998.72 | 977.43 | - | - | - |
| Proteins | g | 52.39 | 52.85 | 49.41 | - | - | - |
| Lipids | g | 40.91 | 37.38 | 35.67 | - | - | - |
| Available carbohydrates | g | 140.24 | 116.20 | 118.33 | - | - | - |
| Oligosaccharides | g | 72.53 | 27.73 | 30.33 | - | - | - |
| Starch | g | 45.80 | 60.68 | 57.63 | 0.000339 | 0.000557 | - |
| Soluble Fiber | g | 1.68 | 1.64 | 1.64 | - | - | - |
| Unsoluble Fiber | g | 5.16 | 4.61 | 4.20 | - | - | - |
| Total Fiber | g | 11.54 | 10.30 | 8.84 | - | - | - |
| Cholesterol | mg | 185.89 | 160.35 | 176.43 | - | - | - |
| SFA | g | 12.96 | 10.68 | 9.84 | - | - | - |
| P-UFA | g | 4.70 | 4.22 | 3.95 | - | - | - |
| M-UFA | g | 13.30 | 11.06 | 10.27 | - | - | - |
| UFA | g | 18.01 | 15.29 | 14.20 | - | - | - |
| Animal Proteins | g | 20.01 | 19.84 | 17.97 | - | - | - |
| Vegetal Proteins | g | 9.58 | 7.65 | 7.17 | - | - | - |
| Minerals Salts | | | | | | | |
| Calcium | mg | 364.45 | 317.25 | 344.07 | - | - | - |
| Sodium | mg | 1478.50 | 1088.66 | 905.57 | - | 0.029933 | - |
| Potassium | mg | 1760.33 | 1465.27 | 1369.72 | - | 0.030477 | - |
| Phosphorus | mg | 733.81 | 662.86 | 619.32 | - | - | - |
| Iron | mg | 7.07 | 7.91 | 6.73 | - | - | - |
| Zinc | mg | 6.44 | 5.90 | 5.85 | - | - | - |
| Magnesium | mg | 91.11 | 87.66 | 80.45 | - | - | - |
| Copper | mg | 0.60 | 0.48 | 0.46 | - | - | - |
| Chlorine | mg | 0.00 | 0.00 | 0.00 | - | - | - |
| Chromium | mg | 0.000002 | 0.000003 | 0.000002 | - | - | - |
| Fluor | mg | 56.09 | 61.46 | 37.53 | - | - | - |
| Iodine | mg | 0.04 | 0.03 | 0.02 | - | 0.036783 | - |
| Manganese | mg | 0.48 | 1.85 | 1.82 | 0.000272 | < 0.000001 | - |
| Molybdenum | mg | 0.003 | 0.003 | 0.003 | - | - | - |
| Selenium | mg | 0.011 | 0.015 | 0.017 | - | - | - |
| Total Mineral Salts | mg | 4498.93 | 3699.37 | 3371.56 | 0.036859 | 0.012665 | - |
| Vitamins | | | | | | | |
| Vitamin B1 or Thiamine | mg | 0.92 | 0.66 | 0.62 | 0.046484 | 0.029001 | - |
| Vitamin B2 or Riboflavin | mg | 0.95 | 1.02 | 0.94 | - | - | - |
| Vitamin B3 or Niacin | mg | 10.71 | 11.50 | 8.70 | - | - | 0.040136 |
| Vitamin B5 or Pantothenic acid | mg | 0.59 | 0.56 | 0.52 | - | - | - |
| Vitamin B6 or Pyridoxine | mg | 1.02 | 1.00 | 0.77 | - | - | 0.042448 |
| Vitamina B8 or Biotin | mg | 0.004 | 0.003 | 0.003 | - | - | - |
| Vitamina B12 or Cobalamin | mg | 0.0005 | 0.0006 | 0.0007 | - | - | - |
| Total Vitamin B group | mg | 14.19 | 14.75 | 11.57 | - | - | - |
| Vitamin A or Retinol | mg | 0.48 | 0.75 | 0.36 | - | - | - |
| Vitamin C or Ascorbic acid | mg | 71.94 | 45.73 | 64.15 | - | - | - |
| Vitamin D | mg | 0.002 | 0.003 | 0.002 | - | - | - |
| Vitamin E or Tocopherol | mg | 4.13 | 3.26 | 2.53 | - | - | - |
| Vitamin K or Naphthoquinone | mg | 0.03 | 0.01 | 0.01 | - | - | - |
| Vitamin M or Folic acid | mg | 0.16 | 0.17 | 0.14 | - | - | - |
| Total Vitamins | mg | 90.92 | 64.66 | 78.77 | - | - | - |
| Alpha-tocopherol | mg | 0.35 | 0.26 | 0.18 | - | 0.049937 | - |
| Essential Amino acids | | | | | | | |
| Lysine | mg | 1793.09 | 1981.80 | 1855.23 | - | - | - |
| Threonine | mg | 1048.21 | 1126.00 | 1083.99 | - | - | - |
| Methionine | mg | 669.86 | 700.25 | 673.68 | - | - | - |
| Isoleucine | mg | 1193.55 | 1262.92 | 1234.67 | - | - | - |
| Leucine | mg | 2188.44 | 2267.13 | 2167.10 | - | - | - |
| Valine | mg | 1397.83 | 1461.40 | 1400.82 | - | - | - |
| Phenylalanine | mg | 1275.44 | 1297.78 | 1211.80 | - | - | - |
| Tryptophan | mg | 301.65 | 304.08 | 293.27 | - | - | - |
| Total Essential Aminoacids | mg | 9868.07 | 10401.38 | 9920.56 | - | - | - |
| Other Amino acids | | | | | | | |
| Histidine | mg | 850.60 | 878.37 | 809.00 | - | - | - |
| Arginine | mg | 1391.27 | 1506.04 | 1467.32 | - | - | - |
| Aspartic acid | mg | 2229.45 | 2385.47 | 2246.52 | - | - | - |
| Serine | mg | 1271.71 | 1328.59 | 1256.97 | - | - | - |
| Glutamic acid | mg | 5620.38 | 5606.53 | 5556.92 | - | - | - |
| Proline | mg | 1913.25 | 1874.25 | 1836.48 | - | - | - |
| Glycine | mg | 1027.05 | 1129.25 | 2711.10 | - | - | - |
| Alanine | mg | 1245.05 | 1344.46 | 1115.33 | - | - | - |
| Cystine | mg | 392.21 | 386.19 | 1279.50 | - | 0.000001 | < 0.000001 |
| Tyrosine | mg | 2921.06 | 1162.63 | 394.99 | - | - | 0.000538 |
| Fatty Acids | | | | | | | |
| C4:0-C10:0 | g | 0.07 | 0.14 | 0.16 | - | - | - |
| C12:0 laurel | g | 0.05 | 0.18 | 0.18 | - | - | - |
| C14:0 myristic | g | 0.26 | 0.33 | 0.34 | - | - | - |
| C16:0 palmitic | g | 2.03 | 1.59 | 1.86 | - | - | - |
| C18:0 stearic | g | 0.93 | 0.69 | 1.06 | - | - | - |
| C20:0 arachidic | g | 0.02 | 0.01 | 0.02 | - | - | - |
| C22:0 behenic | g | 0.002 | 0.00 | 0.002 | - | - | 0.011333 |
| C14:1 miristoleicus acid | g | 0.02 | 0.03 | 0.03 | - | - | - |
| C16:1 palmitoleic acid | g | 0.26 | 0.23 | 0.22 | - | - | - |
| C18:1 oleic | g | 8.04 | 4.54 | 4.77 | 0.021393 | 0.034917 | - |
| C20:1 eicosaenoic | g | 0.17 | 0.12 | 0.07 | - | - | - |
| C22:1 erucic | g | 0.09 | 0.07 | 0.05 | - | - | - |
| C18:2 linoleic | g | 2.45 | 1.68 | 1.84 | - | - | - |
| C18:3 linolenic | g | 0.29 | 0.18 | 0.25 | - | - | - |
| C20:4 arachidonic | g | 0.10 | 0.09 | 0.09 | - | - | - |
| C20:5 EPA | g | 0.13 | 0.13 | 0.07 | - | - | - |
| C22:6 DHA | g | 0.17 | 0.17 | 0.09 | - | - | - |
| Others | | | | | | | |
| Total Polyphenols | mg | 304.45 | 196.56 | 259.27 | - | - | - |
| Beta-carotene | mg | 889.22 | 454.47 | 396.85 | - | - | - |
| Phytic acid | g | 0.17 | 0.15 | 0.15 | - | - | - |
| Oxalic acid | mg | 35.14 | 12.54 | 31.47 | - | - | - |
| Cellulose | g | 1.02 | 0.90 | 1.05 | - | - | - |
| Purine | mg | 29.25 | 39.96 | 38.02 | - | - | - |
| Water | g | 501.97 | 379.76 | 383.97 | 0.040717 | - | - |
| Alcohol | Kcal | 11.83 | 8.15 | 10.31 | - | - | - |
| Edible Portion | g | 574.56 | 424.76 | 409.69 | 0.008445 | 0.005485 | - |
| H-Orac | µmol | 2867.92 | 2107.22 | 2245.90 | - | - | - |
| L-Orac | µmol | 56.49 | 25.28 | 42.10 | - | - | - |
| Total Orac | µmol | 2900.61 | 2118.15 | 2256.43 | - | - | - |
| Pral | pr | 6.51 | 11.09 | 10.79 | - | - | - |

**Supplementary Table S2.** Copy number (CN Log) obtained from qPCR analysis carried out on fecal samples from the three subject cohort subsets: HC, MHO, and MUO. Average values and standard deviations have been reported.

|  | **HC** | **MHO** | **MUO** |
| --- | --- | --- | --- |
| *Bifidobacterium* genus | 10,9 ± 0,3 | 10,1 ± 1,2 | 9,5 ± 1,3 |
| *Bifidobacterium longum* | 9,6 ± 0,3 | 8,8 ± 1,4 | 8,4 ± 1,7 |
| *Bifidobacterium breve* | 7,0 ± 0,2 | 6,9 ± 0,3 | 6,9 ± 0,4 |
| *Bifidobacterium infantis* | 6,1 ± 1,3 | 5,0 ± 0,9 | 4,7 ± 0,4 |
| *Bifidobacterium bifidum* | 8,4 ± 2,4 | 7,1 ± 2,0 | 6,5 ± 1,4 |
| *Bifidobacterium adolescentis* | 11,2 ± 0,3 | 10,5 ± 1,3 | 8,4 ± 1,3 |
| *Lactobacillus* genus | 8,4 ± 0,7 | 8,6 ± 0,4 | 8,6 ± 0,9 |
| *Lactiplantibacillus plantarum* | 7,8 ± 0,4 | 7,9 ± 0,5 | 8,1 ± 0,7 |
| *Lacticaseibacillus rhamnosus* | 6,4 ± 0,6 | 5,9 ± 0,3 | 7,0 ± 1,7 |
| *Limosilactobacillus reuteri* | 0,0 ± 0,0 | 0,0 ± 0,0 | 0,0 ± 0,0 |
| *Limosilactobacillus fermentum* | 6,9 ± 0,9 | 6,7 ± 0,2 | 7,1 ± 0,6 |
| *Clostridium coccoides* group | 10,4 ± 0,2 | 8,5 ± 0,9 | 8,5 ± 0,7 |
| *Clostridium leptum* group | 8,5 ± 0,2 | 8,1 ± 1,1 | 8,5 ± 0,3 |
| *Prevotella* genus | 8,5 ± 1,3 | 8,6 ± 1,1 | 10,1 ± 1,3 |
| *Atopobium* cluster | 9,5 ± 0,5 | 9,4 ± 0,5 | 9,2 ± 1,2 |
| *Bacteroides fragilis* group | 8,4 ± 0,7 | 8,5 ± 0,8 | 8,6 ± 0,6 |
| *Desulfovibrio* genus | 6,2 ± 1,0 | 6,1 ± 1,3 | 7,4 ± 1,1 |
| *Akkermansia muciniphila* | 7,8 ± 0,8 | 7,4 ± 0,8 | 7,1 ± 0,9 |

**Supplementary Figure S1.** Value of BIC versus the number of clusters and DAPC assign plot relative to metabolic clinical parameters. The inflection point (minimum) of the BIC curve indicates how the whole set of variables allowed for potentially divide samples into three possible *a priori* clusters. Right panel) proportions of successful reassignments. Heat colours represent membership probabilities (red=1, white=0). When the *a priori* assigned cluster was respected by the “a posterior” group, a blue cross on a red background was reported.

**Supplementary Figure S2.** Concentration (ppm) of short-chain fatty acids (SCFAs) in fecal samples from healthy (HC), metabolically healthy obesity (MHO), and metabolically unhealthy obesity (MUO) cohorts. The asterisk indicated the only statistically significant comparison (P < 0.05).
